# Establishment of a humanized mouse model using steady‐state peripheral blood‐derived hematopoietic stem and progenitor cells facilitates screening of cancer‐targeted T‐cell repertoires

**DOI:** 10.1002/cai2.118

**Published:** 2024-04-15

**Authors:** Yulin Xu, Wei Shan, Qian Luo, Meng Zhang, Dawei Huo, Yijin Chen, Honghu Li, Yishan Ye, Xiaohong Yu, Yi Luo, He Huang

**Affiliations:** ^1^ Bone Marrow Transplantation Center, The First Affiliated Hospital Zhejiang University School of Medicine Hangzhou China; ^2^ Liangzhu Laboratory Zhejiang University Hangzhou China; ^3^ Institute of Hematology Zhejiang University Hangzhou China; ^4^ Zhejiang Province Engineering Research Center for Stem Cell and Immunity Therapy Hangzhou China; ^5^ School of Medicine Zhejiang University Hangzhou China

**Keywords:** cancer‐targeted T‐cell receptor T (TCR‐T) cells, circulating hematopoietic stem and progenitor cells (HSPCs), humanized mouse model, steady‐state peripheral blood, T‐cell receptor β‐chain (TRB), three‐dimensional culture

## Abstract

**Background:**

Cancer‐targeted T‐cell receptor T (TCR‐T) cells hold promise in treating cancers such as hematological malignancies and breast cancers. However, approaches to obtain cancer‐reactive TCR‐T cells have been unsuccessful.

**Methods:**

Here, we developed a novel strategy to screen for cancer‐targeted TCR‐T cells using a special humanized mouse model with person‐specific immune fingerprints. Rare steady‐state circulating hematopoietic stem and progenitor cells were expanded via three‐dimensional culture of steady‐state peripheral blood mononuclear cells, and then the expanded cells were applied to establish humanized mice. The human immune system was evaluated according to the kinetics of dendritic cells, monocytes, T‐cell subsets, and cytokines. To fully stimulate the immune response and to obtain B‐cell precursor NAML‐6‐ and triple‐negative breast cancer MDA‐MB‐231‐targeted TCR‐T cells, we used the inactivated cells above to treat humanized mice twice a day every 7 days. Then, human T cells were processed for TCR β‐chain (TRB) sequencing analysis. After the repertoires had been constructed, features such as the fraction, diversity, and immune signature were investigated.

**Results:**

The results demonstrated an increase in diversity and clonality of T cells after treatment. The preferential usage and features of TRBV, TRBJ, and the V–J combination were also changed. The stress also induced highly clonal expansion. Tumor burden and survival analysis demonstrated that stress induction could significantly inhibit the growth of subsequently transfused live tumor cells and prolong the survival of the humanized mice.

**Conclusions:**

We constructed a personalized humanized mouse model to screen cancer‐targeted TCR‐T pools. Our platform provides an effective source of cancer‐targeted TCR‐T cells and allows for the design of patient‐specific engineered T cells. It therefore has the potential to greatly benefit cancer treatment.

## Graphical abstract

Cancer‐targeted T‐cell receptor T (TCR‐T) cells hold promise in treating malignancies but with limited source. We applied steady‐state peripheral blood mononuclear cells via three‐dimensional culture to construct humanized mouse model for cancer‐targeted TCR‐T repertoire screening.

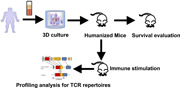

**Abbreviations**
3D: three dimensional
AA: amino acid
CAR‐T: chimeric antigen receptor‐T
CDR3: third complementarity determining region
cHSPCs: circulating hematopoietic stem and progenitor cells
DCs: dendritic cells
MDA‐MB‐231 cells: metastatic breast cancer cells
NAML‐6 cells: leukemic precursor B cells
NCG mice: NOD‐Prkdc^em26Cd52^Il2rg^em26Cd22^/Nju mice
PBMNCs: peripheral blood mononuclear cells
PBS: phosphate‐buffered saline
PCR: polymerase chain reaction
SEM: standard error of mean
TCR‐T: T‐cell receptor T
TRB: TCR β‐chain
TRBJ: J fragments of T‐cell receptor β‐chain
TRBV: V fragments of T‐cell receptor β‐chain

## 1 INTRODUCTION

Cancer severely threatens human life because of its associated poor prognosis, low cure rate, potential for lethal metastasis, and high mortality rate. Traditional cancer treatments, including surgical resection, radiotherapy, and chemotherapy, enhance patient survival but may compromise quality of life and do not always protect against relapse [1, 2]. In recent years, engineered T‐cell immunotherapy, involving chimeric antigen receptor‐T (CAR‐T) cells, has offered promising overcomes in cancer therapy as a result of its low toxicity, good treatment efficacy, and immune system boosting effects [3, 4]. Therefore, immunotherapy offers many advantages as a therapeutic strategy to cure tumors.

Despite promising efficacy in treating hematological malignancies, CAR‐T cells exert limited effects on metastatic breast cancers [4, 5]. The production of CAR‐T cells is based on transducing cancer‐specific recognition fragments as single‐chain variable fragments (scFvs) into T cells. However, the design of effective ligand‐binding domains is a challenge, mainly because the molecular sequences for directing recognition of cancer antigens are difficult to elucidate. The parameters to be considered include affinity, avidity, antigen epitope location, accessibility, and engineered T‐cell cytotoxicity functionality [6]. Therefore, this work is time‐  and labor intensive, limiting the widespread clinical application of CAR‐T cells with the potential to exert oncolytic effects.

It is well established that the third complementarity determining region (CDR3) of T‐cell receptor (TCR) β‐chains (TRBs), the most variable region for antigen recognition and binding, is a vital structure to directly interact with the peptide presented by major histocompatibility complex and is the molecular basis for the recognition of cancer‐specific antigens by T cells [7, 8]. Therefore, the effective functional screening of cancer‐targeted TRB CDR3 repertoires will contribute to identifying T cells with rearrangement sequences responding to specific cancers and will also benefit the discovery of specific cancer‐targeting T‐cell clones [9, 10].

Humanized animal models have been applied successfully in many critical disease studies in the areas of immuno‐oncology, infectious disease, and T‐cell modulation, over recent decades [11, 12, 13, 14]. Among these humanized models, the transplantation of CD34^+^ hematopoietic stem and progenitor cells (HSPCs) derived from bone marrow or umbilical cord blood and the direct transplantation of peripheral blood mononuclear cells (PBMNCs) are the most widely applied approaches [15, 16]. Despite conferring the advantage of long‐term (up to 12 months) robust T‐cell maturation and inflammatory responses, the CD34 humanized mouse model is limited to rare HSPCs, is accompanied by the adverse effects of receiving mobilization agents, and has a reported mobilization failure among patients of 5%–46% [17, 18]. PBMNC‐constructed humanized mice are applicable for short‐term studies offering only T‐cell reconstitution and cannot be used to study the interaction among several immune cells in vivo. Additionally, strategies to select cancer‐targeted TCR‐T‐cell repertoires with personalized immune features have not been reported to date.

We previously reported the development of a three‐dimensional (3D) system using self‐assembly polypeptide Arg–Gly–Asp (RGDs) to efficiently capture and expand rare circulating HSPCs (cHSPCs) in steady‐state peripheral blood without the requirement for mobilization [19]. In that study, the expanded cells conferred the potential for long‐term reconstitution of the human hematopoietic system as T cells, B cells, and myeloid cells. Therefore, steady‐state PBMNCs are a promising resource to construct a humanized mouse model with numerous advantages, including the wide availability and simplicity of this animal model, the noninvasive nature of cell collection, and the patient‐specific targeting of the immune system.

In this study, we applied PBMNC‐derived 3D‐cultured HSPCs to construct humanized mice for screening cancer‐targeted TCR‐T‐cell profiles. With successful reconstitution of human immune systems containing dendritic cells (DCs), monocytes, and T‐cell subsets, the mice were transfused with inactive NAML‐6 and MDA‐MB‐231 twice a day, every 7 days. The kinetics of the immune cells and their secretion cytokines were monitored. Human T cells were then purified and subjected to TRB CDR3 profile construction and analysis. The rearrangement features, diversity, and clonality of the CDR3 profiles revealed a positive response to stimulation. Treatment with inactive cancer cells significantly prolonged the survival time of the mice with xenografts of tumor cells. Our system contributes to the screening of cancer‐targeted TCR‐T cells and to the design of effective ligand‐binding domains as scFv for CAR‐T cell production.

## 2 MATERIALS AND METHODS

### 2.1 Establishment of 3D culture of PBMNCs

The studies involving human participants were reviewed and approved by the Clinical Research Ethics Committee of the First Affiliated Hospital, Zhejiang University School of Medicine. The participants provided their written informed consent to participate in this study. PBMNCs were collected with human peripheral blood lymphocyte isolation solution (Haoyang Biological Technology Co., Ltd) at 400*g* for 25 min, followed by washing with phosphate‐buffered saline (PBS) free of Ca^2+^ and Mg^2+^, 2–3 times. The establishment of a 3D culture of PBMNCs was performed as previously described [19, 20].

### 2.2 Construction of humanized mice using PBMNC‐derived 3D‐cultured HSPCs

The animal study was reviewed and approved by the Animal Experimental Ethical Inspection of the First Affiliated Hospital, College of Medicine, Zhejiang University. After a 10–14‐day culture in the 3D system, the expanded cells were treated with trypsin (0.25%)‐EDTA (0.02%). After washing with PBS and filtration through 40‐µm strainers, ~1 × 10^6^–2 × 10^6^ cells were administered to 7–8‐week‐old NOD‐Prkdc^em26Cd52^Il2rg^em26Cd22^/Nju (NCG) mice irradiated with 1.8 Gray via tibia injection. The types of human immune cells were analyzed by flow cytometry at the indicated times. The recipients, with the detected chimerism of human hematopoietic cells including DCs, monocytes, and T cells, were deemed a successful humanized model for further testing.

### 2.3 Inoculation of humanized mice

To fully simulate the interaction of the immune system in the humanized mice and to investigate the protective effect of immune inoculation on the inhibition of subsequent injected xenograft tumor growth in vivo, we applied two tumor cell lines, a B‐cell precursor leukemia cell line NAML‐6, which specifically targets antigen CD19 and has been successfully introduced into CD19‐targeted CAR‐T cells [12], and a triple‐negative breast cancer cell line MDA‐MB‐231 associated with refractory and high‐mortality cancers. NAML‐6 cells were cultured in RPMI‐1640 medium with 10% fetal bovine serum (FBS), and MDA‐MB‐231 breast cancer cells were maintained in Dulbecco's modified Eagle's medium (DMEM) with 10% FBS. On entering the log phase, the populations were treated with 10 mg/mL mitomycin C to inhibit DNA synthesis and nuclear division for 2–3 h, and the cells were collected; then, 2 × 10^6^ cells were injected into the humanized mice via tail intravenous administration. The booster treatment was administered at 7 days. One week later, live NAML‐6 and MDA‐MB‐231 cells were injected. The initial two doses of NAML‐6 and MDA‐MB‐231 cells were used to stimulate the production of tumor‐specific T cells, and the third dose was used to evaluate the antitumor effects of tumor‐specific T cells.

### 2.4 Establishment of the tumor‐carrying mouse model

Suspensions of 1 × 10^6^ NAML‐6 cells (leukemic precursor B cells) and 1 × 10^6^ MDA‐MB‐231 cells (metastatic breast cancer cells) in 300 μL of PBS were injected into NCG mice via the tail vein. Tumors were then measured by flow cytometry at the indicated time points.

### 2.5 Dynamics of human immune cells before and after inactive cancer cell infusion

All antibodies used for flow cytometry analysis in this study are preceded by h or m depending on their targeting of human or mouse cell types, respectively.

For the detection of human DCs and monocytes, based on hCD45^+^ cells, we first gated hCD3^−^CD19^−^ populations in which hCD14^+^ monocytes were obtained. Furthermore, hHLA‐DR^+^CD11c^+^ myeloid DCs and hHLA‐DR^+^CD123^+^ plasmacytoid DCs were isolated in hCD3^−^CD19^−^CD14^−^ cells.

To detect different subsets of human T cells, hCD3^+^ cells were first gated from the hCD45^+^ population. Then, hCD4^+^CD8^−^ and hCD4^−^CD8^+^ cell subgroups were obtained. At the same time, the proportions of hCD62L^+^CD45RA^+^ naive T cells (Tn), hCD62L^+^CD45RA^−^ central memory T cells (Tcm), hCD62L^−^CD45RA^+^ effector T cells (Te), and hCD62L^−^CD45RA^−^ effector memory T cells (Tem) were obtained on the basis of the hCD3^+^ population.

### 2.6 Human T‐cell isolation and RNA extraction

Human T cells were collected from the humanized mice at the indicated time and purified using the EasySep™ Human CD3 Positive Selection Kit II (STEMCELL Technologies Inc.). The total RNA was extracted using Trizol reagent (Invitrogen) according to the manufacturer's instructions. The integrity of the RNA was tested by analyzing the transcript bands corresponding to 28S and 18S rRNA in a 1.2% agarose gel.

### 2.7 Construction of the TCR sequencing library

The ImmuHub TCR Profiling System was applied at the deep level (ImmuQuad Biotech) for high‐throughput sequencing of TRB. Total RNA was isolated from human CD3^+^ cells isolated from the peripheral blood of humanized mice using the RNeasy Plus Mini Kit (Qiagen). RNA samples were analyzed by NGS for TCRβ. Briefly, a 5′ RACE unbiased amplification protocol was used. One common forward adaptor primer and one reverse primer corresponding to the constant (C) regions of each of the TCRβ were designed to facilitate PCR amplification of cDNA sequences in a less biased manner. This protocol uses unique molecular identifiers introduced in the course of cDNA synthesis to control bottlenecks and to eliminate PCR and sequencing errors.

A NovaSeq® system with PE150 mode (Illumina) was used for purified PCR product sequencing. According to unique molecular barcode adapters, PCR duplicates and low‐quality sequences were filtered from the raw data. Segments of the V, D, J, and C genes were mapped to the reference sequences of the international ImMunoGeneTics information database (http://www.imgt.org), and then CDR3 regions were extracted and assembled for clones. The resulting nucleotide and amino acid sequences of CDR3 of TCRβ were determined and those with out‐of‐frame and stop codon sequences were removed from the identified TCRβ repertoire. We further defined amounts of each TCRβ clonotype by adding numbers of TCRβ clones sharing the same nucleotide sequence of CDR3.

### 2.8 Clonality signature analysis for TRB CDR3 repertoires

The datasets of FASTA sequences were submitted to IMGT/highV‐QUEST for gene interpretation to construct cancer‐targeted TRB CDR3 repertoires. The information obtained for each clone included the clone count, clone fraction, CDR3 gene sequence, CDR3 amino acid (AA) sequence, and the sequences of the V, D, J, and C segments. TRB CDR3 repertoires were analyzed for similarities and differences among the groups. Clonotypes and counts were analyzed for diversity and clonal proliferation, respectively. By comparing the AA sequence of CDR3 among the groups, we calculated the total amount for each clonotype. We also analyzed the increase in TRB CDR3 clonotypes and their frequency of changes among the clone distribution in the groups. To investigate new clones produced under immune stimulation conditions, the emerging clonotypes were also analyzed in terms of counts and frequencies.

### 2.9 Statistical analysis

Statistical analyses were performed using Prism software, version 9.0 (GraphPad Software Inc.). The mean ± SEM was used to describe the data with a normal distribution. *p* values were calculated using ordinary one‐way ANOVA or a Student's *t*‐test. The Kolmogorov–Smirnov test was used for comparing the cumulative count and the frequency of dominant clonotypes in the groups. *p*  <  0.05 was considered statistically significant.

## 3 RESULTS

### 3.1 Successful construction of humanized mice using steady‐state peripheral blood mononuclear cell‐derived HSPCs via 3D culture

**Figure 1:**
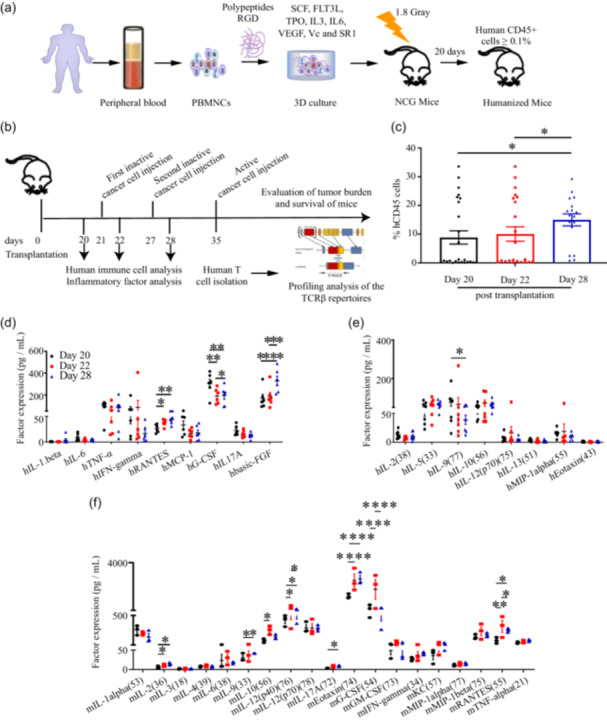
Construction of humanized mice using three‐dimensional (3D)‐cultured steady‐state peripheral blood‐derived cells and in vivo inflammatory factor secretion analysis. (a) Schematic illustration of the expansion of circulating hematopoietic stem and progenitor cells (cHSPCs) in peripheral blood mononuclear cells (PBMNCs) using 3D culture. (b) Schematic of the design used to screen cancer‐targeted T cell repertoires in the constructed humanized mice. (c) Dynamic detection of human CD45 cells at Day 20 (before the first infusion), 22 (after the first infusion), and 28 (after the second infusion). The results showed that human CD45^+^ cells were produced and increased significantly after the second infusion. *N* = 16–27. (d–f) Dynamic changes in human‐ and mouse‐derived inflammatory factor secretion. The results showed that the expression of human‐derived G‐CSF was significantly downregulated, while the levels of bFGF and IL‐9 were significantly upregulated after immune infusion. The expression of other factors, such as TNF‐α and IL‐2, did not change significantly. However, the level of mouse‐derived G‐CSF was significantly upregulated under immune infusion. Chemokine eotaxin also increased significantly. *N* = 6–7 (human‐derived cytokine analysis). *N* = 3 (mouse‐derived cytokine analysis). *p < 0.05, **p < 0.01, ***p < 0.001, ****p < 0.0001.

The scheme for 3D culture is shown in Figure 1a and is consistent with our previous report [19]. PBMNCs isolated from steady‐state peripheral blood were subjected to 3D culture with hematopoietic factors for ~10–14 days. NCG mice irradiated with a sub‐lethal dose of 1.8 Gy were transplanted with 1 × 10^6^–2 × 10^6^ 3D‐derived cells intrafemorally. At Day 20 post‐transplantation, peripheral blood was collected for human chimerism detection. Mice with percentages of human CD45^+^ cells ≥0.1% were considered to have been successfully reconstituted and were applied in further experiments, as illustrated in Figure 1b.

### 3.2 Kinetics of human CD45^+^ cells and cytokine secretion revealed an active response to stress stimulation

At Days 20, 22, and 28 post‐transplantation (i.e., before and after the first and second immune stimulation injections), we collected peripheral blood from the humanized mice via the tail vein and monitored the dynamic changes in human chimerism (Figure 1c). The results showed that human CD45^+^ cells at Day 28 had increased significantly compared with the ratios at Days 20 and 22, indicating the positive response of human immune cells to the second injection.

We also detected the kinetics of human (h) and mouse (m) cytokine secretion during the immune stimulation process using human and mouse cytokine array kits, respectively (Figure 1d–f). The results demonstrated that the expression levels of hRANTES, hG‐CSF, hbasic‐FGF (bFGF) (Figure 1d), and hIL‐9 changed significantly (Figure 1d,e). The levels of mIL‐2, mIL‐9, mIL‐10, mIL‐12 (p40) (76), mIL‐17A, mG‐CSF, mEotaxin, and mRANTES also changed significantly (Figure 1f). Interestingly, the level of hG‐CSF decreased significantly at Days 22 and 28, whereas mG‐CSF levels increased significantly throughout (Figure 1d–f). The levels of both hRANTES and mRANTES were significantly increased at Days 22 and 28 compared with Day 20. Additionally, the level of mIL‐2 increased at Days 22 and 28, indicating the positive response of mouse immune cells to treatment. Furthermore, mIL‐10 significantly increased at Day 22, and mIL‐9 significantly increased at Day 28 (Figure 1f). By contrast, mIL‐6, mGM‐CSF, mMIP‐1α, mMIP‐1β, and mTNF‐α showed little change throughout. We speculated that this may be because humanized mice produce some inflammatory factors during graft‐versus‐host disease.

The kinetics of human and mouse cytokine secretion revealed the active response of the immune system to stress stimulation.

### 3.3 Human dendritic cells and monocytes undergo considerable changes during the stimulation process

In humanized mice, human DCs, monocytes, and T‐cell activity have been reported to support the human immune stress response. We therefore selected these three cell types to analyze the functional effects of inactive cancer cell inoculation.

**Figure 2:**
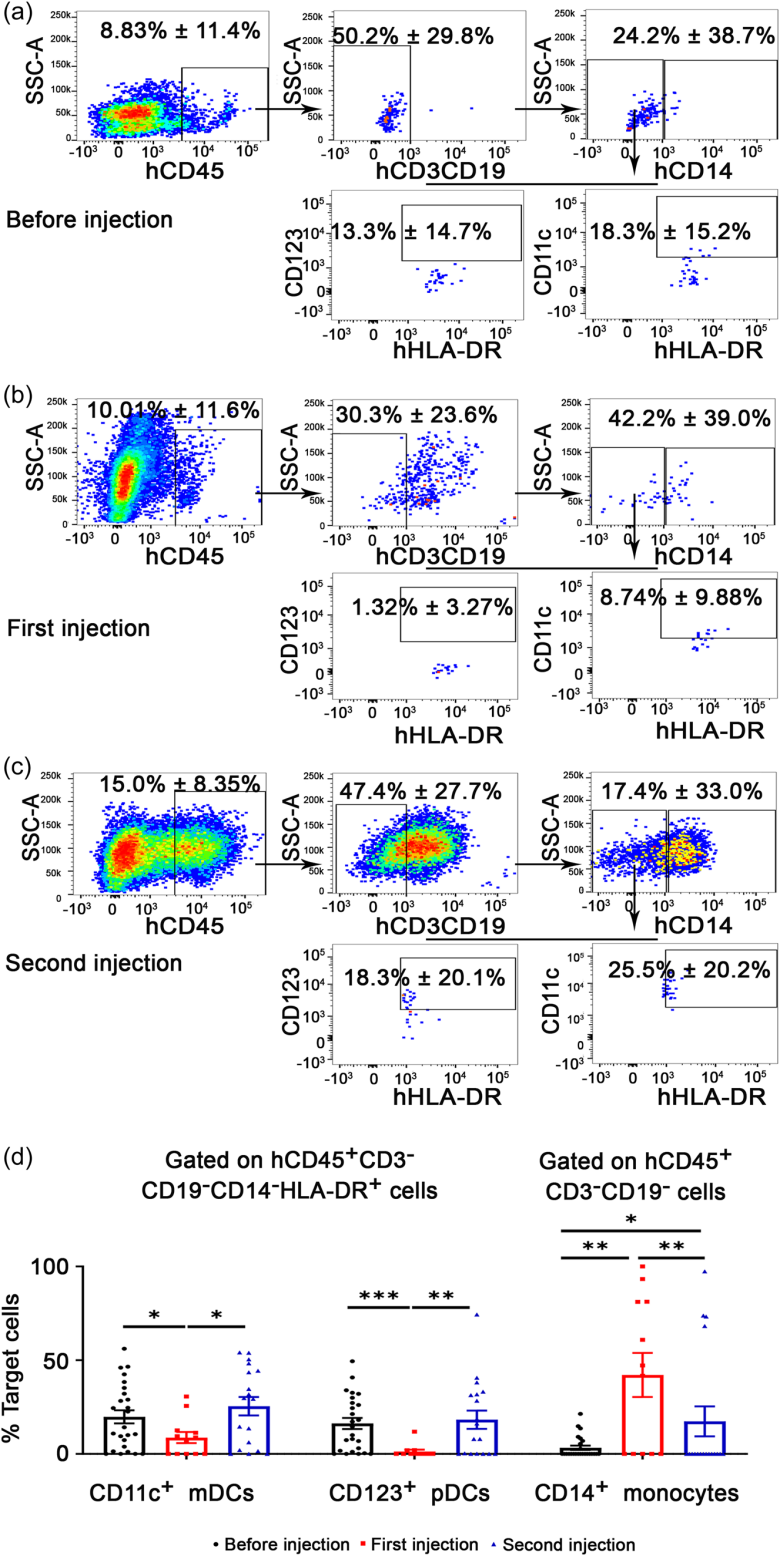
Dynamics of human dendritic cells and monocytes in the humanized mice. (a–c) By gating human CD45^+^ cells, we first isolated CD3^−^CD19^−^ cells. We then further analyzed the frequency of CD3^−^CD19^−^CD14^+^ monocytes and detected the expression levels of human CD3^−^CD19^−^CD14^−^HLA‐DR^+^CD11c^+^ myeloid DCs and CD3^−^CD19^−^CD14^−^HLA‐DR^+^ CD123^+^ plasmacytoid DCs (a) before infusion, (b) after the first infusion, and (c) after the second infusion. (d) Statistical data were calculated for the dynamic expression of human DCs and monocytes in the humanized mice. The results showed that human CD45^+^CD3^−^CD19^−^CD14^−^HLA‐DR^+^CD11c^+^ myeloid DCs and CD45^+^CD3^−^CD19^−^CD14^−^HLA^−^DR^+^CD123^+^ plasmacytoid DCs decreased significantly after the first immune infusion, while they increased after the second infusion, to levels equivalent to those before infusion. Interestingly, CD14^+^ monocytes were continuously significantly upregulated after the first and second infusions, especially after the first infusion. These results indicated that inactive cancer cell infusion particularly affected DCs, including myeloid and plasmacytoid DCs, and also regulated the expression of CD14^+^ monocytes. *N* = 12–25. *p < 0.05, **p < 0.01, ***p < 0.001.

Based on human CD45^+^ cells, we gated the CD3^−^CD19^−^ population, which was further grouped into CD14^+^ monocytes (CD45^+^CD19^−^CD3^−^CD14^+^ monocytes) and CD14^−^ cells. Among CD14^−^ cells, HLA‐DR^+^CD11c^+^ cells were identified as myeloid DCs (CD45^+^CD19^−^CD3^−^CD14^−^HLA‐DR^+^ CD11c^+^ myeloid DCs, mDCs), and HLA‐DR^+^CD123^+^ cells were identified as plasmacytoid DCs (CD45^+^CD19^−^CD3^−^CD14^−^HLA‐DR^+^ CD123^+^ plasmacytoid DCs, pDCs) (Figure 2). Flow cytometric analysis of mDCs and pDCs before infusion, and after the first and second infusions, of inactive cancer cells was conducted (Figure 2a–c). The dynamic changes in mDCs, pDCs, and monocytes were statistically calculated (Figure 2d). The results demonstrated that the levels of mDCs and pDCs were relatively high before infusion, decreased after the first infusion, and then increased significantly after the second infusion, while CD14^+^ monocytes showed the opposite trend to DCs. It is well‐established that monocytes and DCs play an important role in antigen presentation and further activation of T cells. In this study, the expression levels of CD14^+^ monocytes were low initially, whereas mDCs and pDCs accounted for ~15% of CD45^+^CD19^−^CD3^−^CD14^−^HLA‐DR^+^ cells. After the first infusion, CD14^+^ monocytes were significantly upregulated, indicating a strong response to the exogenous infusion of inactive cancer cells. By contrast, the proportions of mDCs and pDCs were significantly downregulated to ~8% and ~1%, respectively, in the CD45^+^CD19^−^CD3^−^CD14^−^HLA‐DR^+^ population. After the second infusion, CD14^+^ monocytes were again significantly upregulated compared with the levels before infusion, indicating that they might continue to respond to exogenous infusion, although not as strongly as to the first infusion. The proportions of mDCs and pDCs in CD45^+^CD19^−^CD3^−^CD14^−^HLA^−^DR^+^ cells were not significantly changed compared with before infusion, but were significantly higher than those after the first infusion.

These results suggested that CD14^+^ monocytes responded strongly to immune stimulation, while mDCs and pDCs were suppressed following the first infusion. The kinetic changes in human monocytes and DCs revealed the active response of the immune system to inactive cancer cell treatment in humanized mice.

### 3.4 Various human T‐cell subsets showed different dynamic changes after treatment

**Figure 3:**
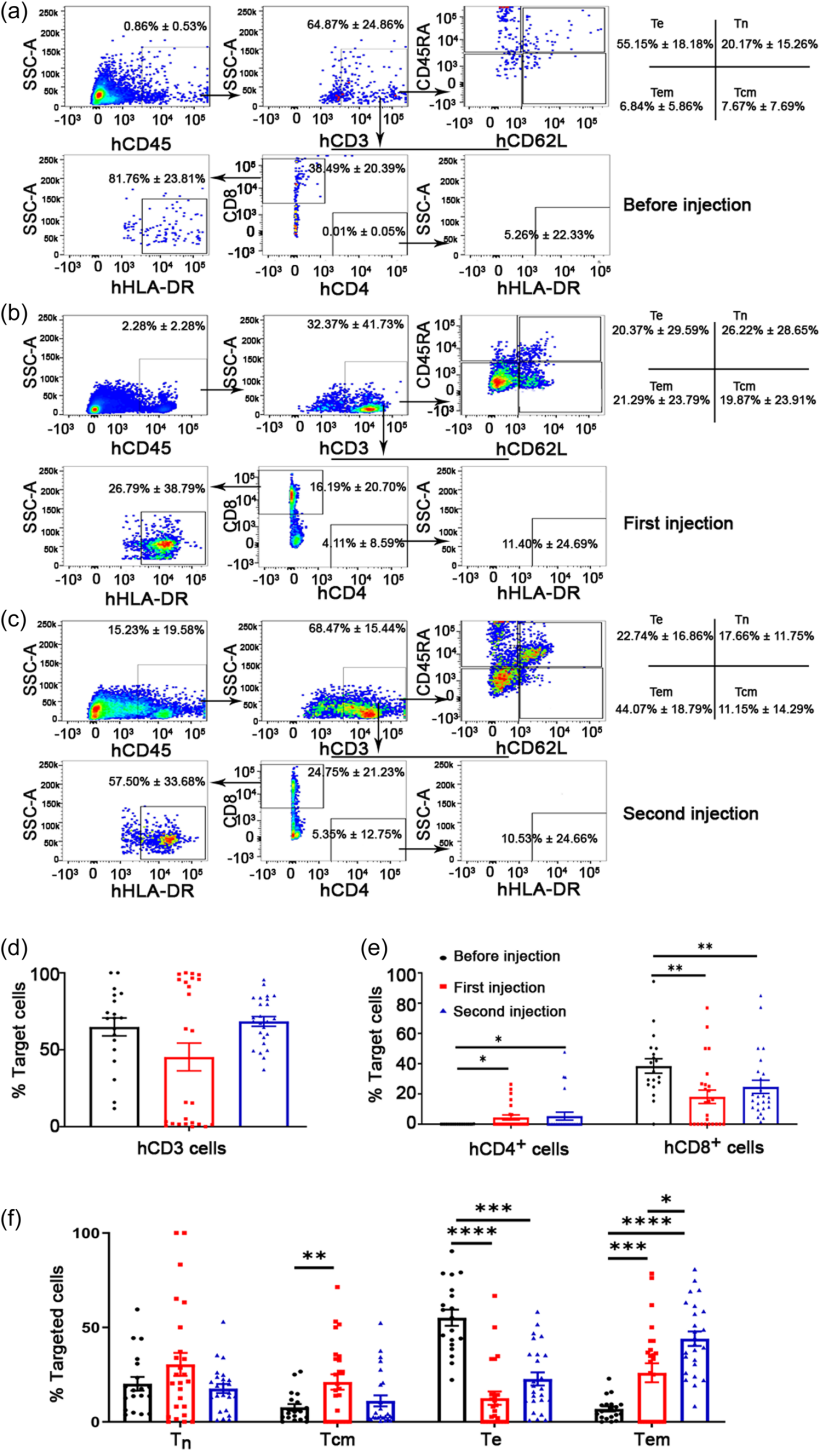
Dynamic detection of human T lymphoid subsets in humanized mice upon cancer cell infusion stimulation. (a–c) Based on the human CD45^+^ cell population, we first gated the frequency of human CD3^+^ cells. In the hCD3‐positive profile, we applied human hCD4, hCD8, and hHLA‐DR to detect the kinetics of human T helper cells and T cytotoxic subpopulations in their activated state. Additionally, according to the CD3^+^ population, we determined the change in the ratio of functional T cells, such as hCD62L^+^CD45RA^+^ naive T cells (Tn), hCD62L^+^CD45RA^−^ central memory T cells (Tcm), hCD62L^−^CD45RA^−^ effector memory T cells (Tem), and hCD62L^−^CD45RA^+^ effector T cells (Te). (d) Statistical analysis of the kinetic populations of human T cells in humanized mice under inactive cancer cell infusion. The results demonstrated that PBMNC‐derived 3D‐cultured cells showed a relatively high population of T leukocytes in hCD45^+^ cells (Figure 3D). (e) Statistical analysis of subtype changes in human T cells, such as CD3^+^CD4^+^ T helper cells and CD3^+^CD8^+^ T cytotoxic cells. The results showed that T helper cells increased after the first and second infusions, T cytotoxic cells decreased after the first and second infusions, and the CD8^+^ HLA‐DR^+^ regulatory T cell subset also decreased, but the levels were higher after the second infusion compared with the first infusion. (f) Statistical analysis of the data demonstrated that Tcm increased significantly after the first infusion, but there was no significant change after the second infusion. Tn and Tem increased significantly. Te decreased significantly after both treatments. *N* = 19–25. *p < 0.05, **p < 0.01, ***p < 0.001, ****p < 0.0001.

By gating the hCD45^+^ population, we further applied human hCD3, hCD4, hCD8, hHLA‐DR, hCD45RA, and hCD62L to detect the kinetics of human T‐cell subtypes. The gating strategies for T‐cell populations were based on the hCD3 profile, and then T‐cell subpopulations were further assessed by their expression of hCD62L and hCD45RA as follows: hCD62L^+^CD45RA^+^ naive T cells (Tn), hCD62L^+^CD45RA^−^ central memory T cells (Tcm), hCD62L^−^CD45RA^−^ effector memory T cells (Tem), and hCD62L^−^CD45RA^+^ effector T cells (Te) (Figure 3a–c). The cohort of several subimmune T‐cell members showed measurable levels in the peripheral circulation. At the same time, we identified hCD4^+^ T helper cells and a hCD8^+^ T cytotoxic subpopulation in the hCD3 profile with doublet exclusion, and we further detected hHLA^−^DR expression in both T subtypes (Figure 3a–c). The statistical data are shown in Figure 3d–f. Consistent with our prior reports, the mice transplanted with PBMNC‐derived 3D‐cultured cells showed a relatively high population of T leukocytes in hCD45^+^ cells (Figure 3d).

CD3^+^CD4^+^ T helper cells increased after the first and second infusions, CD3^+^CD8^+^ T cytotoxic cells decreased after the first and second infusions, and the CD8^+^ HLA‐DR^+^ regulatory T cell subset also decreased but after the second infusion showed an upward trend (Figure 3e).

hCD62L^+^CD45RA^+^ Tn and hCD62L^+^CD45RA^−^ Tcm cells increased significantly after the first infusion, but there was no significant change after the second infusion. hCD62L^−^CD45RA^−^ Tem cells increased significantly after the first and second infusions, while hCD62L^−^CD45RA^+^ Te cells decreased significantly after both treatments (Figure 3f). A potential explanation may be that samples were collected 1 day after immune infusion. In the future, it will be necessary to collect additional data at 4, 8, and 12 h time points after immune infusion to analyze in greater detail the dynamic changes in T cells, which may aid the interpretation of these data.

### 3.5 TRB sequencing profile analysis demonstrated an increase in TCR‐T cell repertoire diversity after inactive cancer cell treatment

Two groups of humanized mice engineered with 3D‐cultured PBMNCs from two volunteers were used for TRB sequencing analysis. The first group was established with PBMNCs from a female (#1 humanized mice), and the second group was established with PBMNCs from a male (#2 humanized mice). Each group comprised ~20 mice, which were further divided into treated and untreated subgroups. The mice receiving inactive cancer cell stimulation were termed the treated group, and those receiving no such injection were termed the untreated group. Information on the samples used for TRB sequencing analysis is provided in Table S1. Human T lymphocytes were purified with the EasySep™ Human CD3 Positive Selection Kit (STEMCELL Technologies Inc.) and then subjected to TRB sequencing analysis.

**Figure 4:**
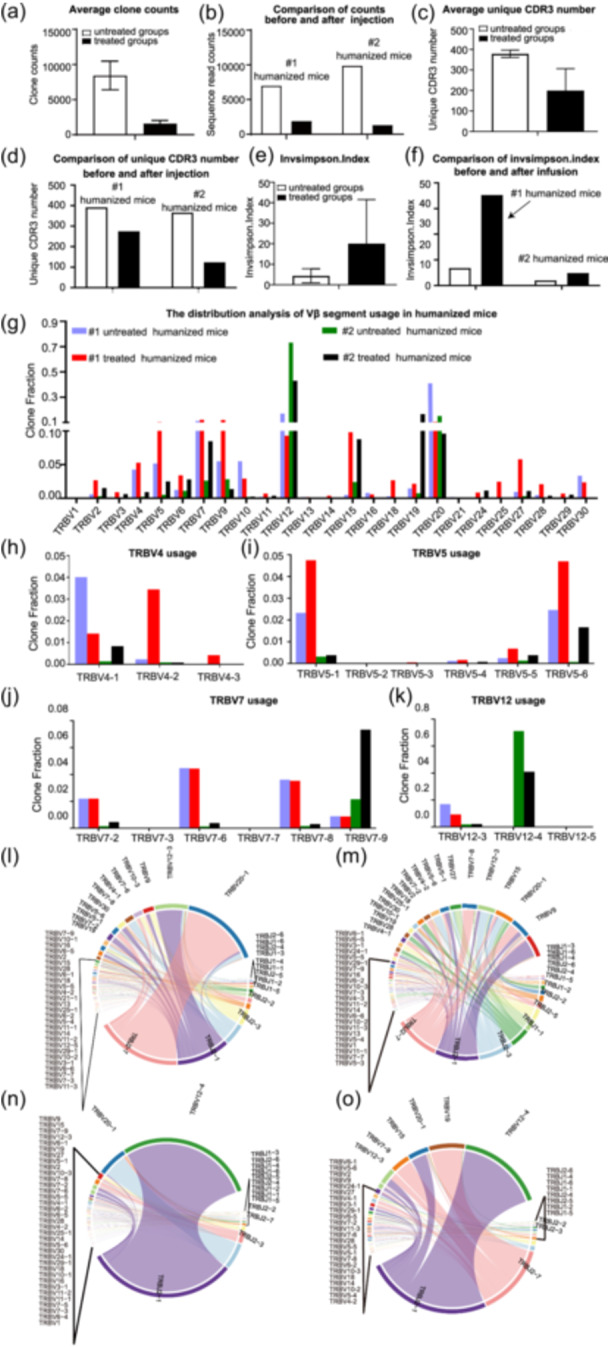
Inactive cancer cell infusion stimulated a characteristic change in human TCR β‐chain CDR3 sequences in humanized mice. (a) Data summary of the clonal changes after quality control for TRB CDR3 repertoires. The average clone number was 8440 in the untreated group, compared with 1619 in the treated group. (b) The counts for each sample were 7002 and 9878 for each untreated humanized mouse and 1916 and 1322 for each treated humanized mouse. (c) Average unique CDR3 numbers in humanized mice. (d) Comparison analysis of the unique CDR3 repertoires in each group. (e) InvSimpson index analysis showed that the clonal diversity decreased after stimulation. (f) The clonal index comparison was analyzed for each sample. The results indicated that the inoculation treatment resulted in an increase in TRB CDR3 clonal diversity. (g) The distribution analysis of TCR β‐chain V fragments (TRBVs) usage in humanized mice. (h–k) Clonal fraction distribution of TRBV4, 5, 7, 12, and their subfamilies. (l–o) Data summary of TRBV, TRBJ gene rearrangement patterns, and the V–J joint usage frequency in each repertoire of the humanized mice.

After high‐throughput sequencing of the TRB regions, we filtered out the low‐quality sequences from the raw data and then mapped the sequences to the reference sequences of the international ImMunoGeneTics information database (http://www.imgt.org). TRB profiles were generated comprising V, D, J, and C gene segments and the corresponding AA sequences with out‐of‐frame sequences and stop codon sequences removed. The average clone count for samples in the untreated groups was 8440, compared with 1619 in the treated groups (Figure 4a). The count for each sample was 7002 for the untreated #1 humanized mice and 9878 for the untreated #2 humanized mice, compared with 1916 for the treated #1 humanized mice and 1322 for the treated #2 humanized mice (Figure 4b). Unique CDR3 analysis revealed that each sample expressed different numbers of unique complementarity determining nucleotide sequences depending on the mice, as follows: 390 for untreated #1 humanized mice, 364 for untreated #2 humanized mice, 273 for treated #1 humanized mice, and 122 for treated #2 humanized mice (Figure 4c,d).

We used the InvSimpson index as the clonality index to quantitatively analyze the expansion of TCR‐T cell clones. The diversity index showed differences as indicated by the InvSimpson index (Figure 4e,f). The clonality index was raised from 6.80 and 1.94 in the untreated groups to 35.3 and 4.86 in the treated groups, respectively. In contrast to the Shannon–Weaver index, the InvSimpson index is a diversity index emphasizing high‐frequency reads (i.e., numerous unique reads). Therefore, our data indicated that the inactive cancer cell treatment produced higher diversity of TCR‐T cell repertoires with high‐frequency reads.

### 3.6 Assays of the preferential usage, frequency, and features of TRBV, TRBJ, and the V–J combination indicated a change in TRB following inactive cancer cell treatment

All fragments of the V‐gene (TRBV), J‐gene (TRBJ), and the V–J gene combination for each sample were obtained via IMGT database comparisons.

We first assessed the effect of immune stimulation on the preferential usage and frequency of TRBVs for each humanized mouse group (Figure 4g and Table S2). TRBV families were classified into 25 families and 41 subfamilies among the groups (Table S3). TRBVs such as V4, V5, V7, V12, and V20 were highly expressed in the subjects, and many of these TRBVs contained a number of subfamilies. The usage of some TRBV subsets is shown in Figure 4h–k.

In the treated #1 humanized mice, the top five ranked TRBVs were TRBV9 (11.9%), TRBV20‐1 (10.5%), TRBV15 (9.81%), TRBV12‐3 (9.29%), and TRBV7‐8 (7.62%), accounting for 49.04% of the total clones. By contrast, in the untreated #1 humanized mice, the top five ranked TRBVs were TRBV20‐1 (40.9%), TRBV12‐3 (16.9%), TRBV9 (~5.50%), TRBV10‐3 (4.61%), and TRBV7‐6 (4.47%), accounting for 72.4% of the total clones. As for the rearrangement kinetics of TRBV–Js, TRBVs were mainly joined with TRBJ2‐7 (23.8%), TRBJ2‐1 (23.0%), and TRBJ2‐3 (18.2%). In the untreated subjects, the corresponding companions were TRBJ2‐7 (43.5%), TRBJ2‐1 (28.1%), and TRBJ2‐3 (10.8%) (Figure 4l,m).

In the treated #2 humanized mice, the top five ranked TRBVs were TRBV12‐4 (40.9%), TRBV19 (16.6%), TRBV20‐1 (9.61%), TRBV15 (8.77%), and TRBV7‐9 (7.33%), accounting for 42.3% of the total clones. By contrast, in the untreated #2 humanized mice, the top five ranked TRBVs were TRBV12‐4 (71.2%), TRBV20‐1 (15.2%), TRBV9 (2.81%), TRBV15 (24.1%), and TRBV7‐9 (2.16%), accounting for 93.7% of the total clones. Compared with the untreated group, the percentage of TRBJ2‐1 decreased from 77.2% to 58.9%, TRBJ2‐7 increased from 5.78% to 33.7%, and TRBJ2‐3 decreased from 13.3% to 1.97% (Figure 4n–o). The composition of the highly expressed V‐gene and J‐gene segments and their corresponding AA sequences are listed in Table S2, along with the highly expressed V–J segment combination (frequency > 0.1% for both groups).

According to the results regarding the preferred TRBVs, TRBJs, and TRBV–J combination, many types of TRBVs were found in both humanized subjects; however, the TRBV12 and TRBV20‐1 families were the main fragments used in humanized mice. Furthermore, TRBJ2‐1 was the main TRBJ family found in each subject. The treated group showed similar usage of TRBVs, TRBJs, and the TRBV–J combination, indicating that the preference for usage (i.e., favoring the TRBV–J rearrangement) is similar in response to the same antigen.

Additionally, based on the results of comparative analysis, we found that some features of TRBV, TRBJ, and their combination, were maintained, with the preferential usage of the TRBV–J rearrangement, although the corresponding percentages varied greatly. Importantly, new TRBV segments and their combinations with TRBJ (such as TRBV5‐3, TRBV1, TRB5‐3‐TRBJ2‐1) were only detected in the treated groups, possibly as a result of the immune cell response. This may prove important in the design of engineered T cells for targeting specific tumors, potentially involving scaled‐up TRBV–J rearrangement segments with distinct V–J usage.

### 3.7 Clonal overlap evaluation revealed higher CDR3 diversity after inactive cancer cell treatment

**Figure 5:**
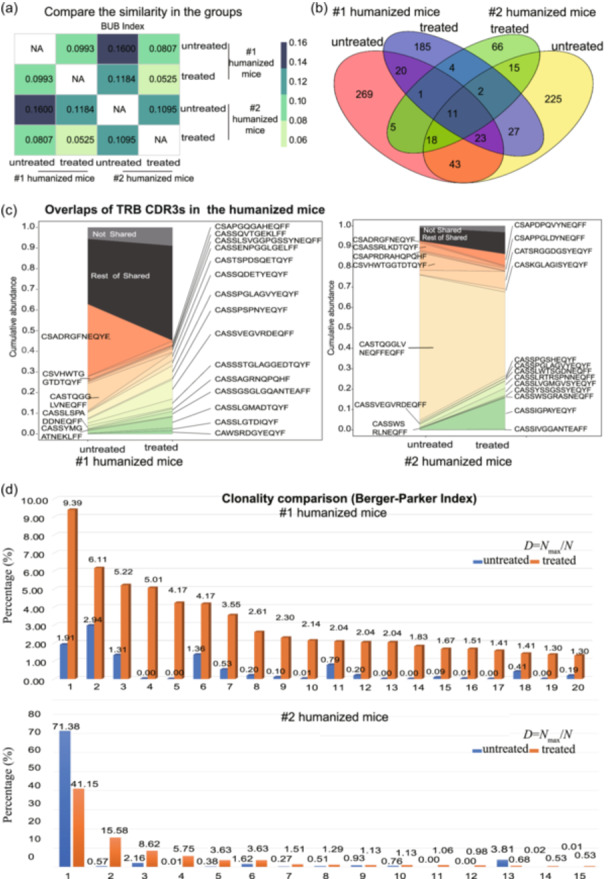
Comparison of the clonality features of the TRB CDR3 repertoires. (a) Similarity among the groups was demonstrated using the Baroni–Urbani and Buser (BUB) index and a heatmap. The darker the color, the closer the number is to 1, and the higher the similarity of the samples. (b) Venn diagram analysis showed the number of shared clones among TRB CDR3 repertoires. (c) Cumulative abundance of the amino acid TRB CDR3 overlap ratio is illustrated based on the 20 most highly represented clonotypes from each subject. (d) Dominant clones were demonstrated with the Berger–Parker index to reveal the diversity of TRB CDR3 sequences. The results showed that the dominant shared clones in the treated group were significantly expanded under immune infusion.

Though alignment comparisons of our high‐throughput sequence datasets to the immune repertoire, we calculated the similarity among the TRB profiles using the Baroni–Urbani and Buser (BUB) index, and the results are presented as a heatmap (Figure 5a). The darker the color, the closer the number to 1, indicating greater similarity between the samples. The results indicated that the samples from mice in the untreated groups showed more similarity than those from mice sharing the same donor. Additionally, the treated groups showed great disparity, indicating that cancer cell inoculation had a significant effect on the range of clonotypes, leading to diversity of TCR‐T cells.

We further performed shared clonotype analysis to identify cancer‐responsive TCR‐T cells produced via a natural response mechanism in vivo. Venn diagram analysis revealed 11 overlapping clonotypes among the groups, with a total of 914 clones (Figure 5b). The #1 humanized mice, generated from female PBMNCs, shared 9.05% (55/608) of clones between the treated and untreated samples, and #2 humanized mice, generated from male PBMNCs, shared 10.5% (46/440) of clones.

Overlap analysis of TRB CDR3 repertoires in the treated and untreated humanized mice suggested that TCR‐T cells were distinct, with only low‐frequency overlap among the groups. Cancer cell inoculation had a considerable effect on the diversity of TCR‐T cells, and therefore, our strategy provided the opportunity to screen for critical cancer‐responsive TRB CDR3 sequences that may then be applied to the design of engineered T cells as CAR‐T cells.

### 3.8 Analysis of the most frequently shared clonotypes uncovered a common TRB profile after inactive cancer cell treatment

To monitor the change in TRB repertoire following inactive cancer cell treatment, we investigated the TRB profiles to assess the top 15–20 high‐frequency overlapping clones and their expansion in each of the hCD45^+^ compartments (Figure 5c,d).

First, we tracked the frequency of the top 20 significant overlaps in the groups (Figure 5c). We observed that the highest frequency clones in #1 humanized mice increased from 1.91% to 9.39%, with a nucleotide sequence of TGTGCCAGCAGCGTAGAAGGGGTTCGGGATGAGCAGTTCTTC. However, the highest frequency clones in #2 humanized mice decreased from 71.38% to 41.15%, with a nucleotide sequence of TGTGCCAGCACCCAGGGTGGTTTGGTCAATGAGCAGTTCTTC. The frequencies of the other top 20 clones in the two groups of humanized mice also changed greatly (Figure 5c,d). Additionally, a few clones were enriched after treatment, implying that the stimulation resulted in a positive T‐cell response in the host.

The clonal proliferation index, the Berger–Parker index, of the top 20 overlapping clones for multiple samples was counted and plotted (Figure 5d). For the #1 humanized mice, only ~10% of clones overlapped between two compartments in the TCRB CDR3 repertoires. Similar tendencies were observed in #2 humanized mice. These results suggested that only a small fraction of clones persistently accumulated in the treated samples.

In addition, we examined the variability of the TCR repertoires among individual subjects. We observed that many of the top 20 clones in one sample did not exist or existed at a low frequency in the other profiles. Moreover, the frequency of shared clones in each repertoire was variable among the groups (0.01%–71.38%, mean = 0.92%) (Table S4). This trend showed that even with the same treatment, the resulting clones differed, which might be related to the specific immune environment in vivo, such as different peptide segments being presented by antigen‐presenting cells, such as monocytes and DCs.

### 3.9 Clonal proliferation analysis demonstrated that dominant clones were produced from neoantigen induction under stress conditions

**Figure 6:**
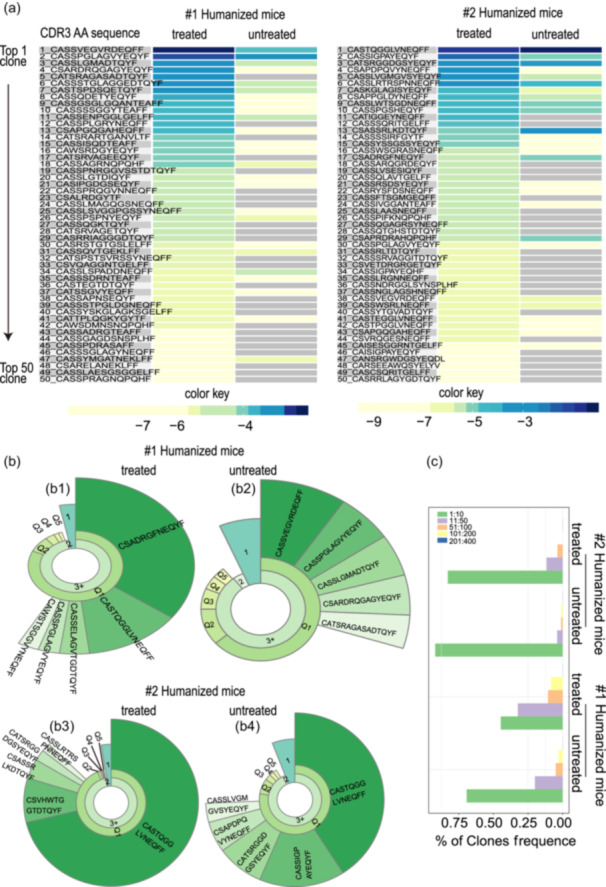
Clone tracking and proliferation of CDR3 in humanized mice upon immune infusion. (a) Top 50 clones and their frequencies were determined using clone‐tracking analysis with a heatmap to reveal the origin of dominant clones in the treated groups. The corresponding amino acid sequences of CDR3 are also listed. A color key was applied to mark the frequency of T‐cell clones: blue indicates higher frequency, yellow indicates lower frequency, and gray indicates not applicable or not detectable. (b) Inactive cancer cell treatment stimulated clonal proliferation of T cells in humanized mice. Representative donut charts of TRB CDR3 clones displayed the clonal expansion in #1 humanized mice (b1 and b2) and #2 humanized mice (b3 and b4) following immune infusion. The fan‐shaped area indicates the corresponding clonal frequency. The radians of 1, 2+, and 3+ represent the total frequency of CDR3 with 1, 2, and 3 or more reads, respectively, in the profiles. Q1–Q5 in the second layer represents the clonal frequencies of T cells per 20% (from high to low). Amino acid sequences are shown for the top five clones in the CDR3 repertoires, with frequencies indicated by the radian of the shape. The larger the radian, the higher the frequency of expansion of a specific TCR clone. The area of “1”: the summed frequencies of T cell clones that only had one sequence read. The area of “2+”: the summed frequencies of T cell clones that had more than two sequence reads. The area of “3+”: the summed frequencies of T cell clones that had more than three sequence reads. The proportion of “1” and “2” is an important indicator of T cell diversity. (c) In each sample, the top 10 clones accounted for more than 50%.

We traced the expanded clones induced in the cancer‐inoculation‐treated groups back to the untreated group. We identified 218 and 76 new clones in the #1 and #2 humanized mice, respectively (Table S5). The top 50 prevalent T‐cell clonotypes in the treated groups were selected and tracked back using their frequencies to their initial companions. As displayed in the heatmap, the top 50 T‐cell clones dominating in the treated groups were not detected or were detected at low frequency in the untreated groups (Figure 6a), implying that cancer infusion stimulation treatment produced new T‐cell clonotypes, and further promoted their preferential expansion, therefore becoming the dominant clones in the treated groups.

Next, we compared the total frequency of the top 20 TRB CDR3s in the treated groups to their initial total frequency in the untreated groups. For #1 humanized mice, the total frequency of the top 20 TRB CDR3 clones was 3.06% ± 0.02% in the treated groups and 0.5% ± 0.008% in the untreated samples (mean ± SEM) (Figure S1; *p* < 0.0001). For #2 humanized mice, the corresponding numbers were 4.44% ± 0.09% and 4.15% ± 0.15%, respectively (*p* = 0.9447), which might reflect the fact that one clone accounted for more than 70% of the total clones in the original sample. The results described above further indicated that most dominant clones in the treated groups were produced from the exclusive response to cancer cell inoculation rather than expansion of the originally endogenous untreated T‐cell pool. An additional explanation might be the limitations of sequencing detection.

### 3.10 Cancer cell inoculation induced highly clonal expansion of T‐cell clones in the humanized mice

We found that TCR diversity increased in the treated groups. As shown in Figure 6b, the first layer of “1” “2” and “3” presents the percentage of T cells possessing 1, 2, or more than three clones, respectively. The proportions of “1” and “2” were important indicators of T‐cell diversity. Q1–Q5 in the second layer represent the rate of every 20% of T‐cell clones (frequency from high to low). The third layer presents the AA sequence of the CDR3 region of the top five TCR‐T cell clones. The wider the area of the layer, the higher the clonal expansion of a specific TCR‐T cell clone.

According to the cloning frequency, each sample possessed dominant clones, with the top 10 clones accounting for more than 50% of the total clones (Figure 6c), indicating that each host harbors prevalent T‐cell clones to maintain the immune balance of the body under specific conditions.

### 3.11 Nucleotide and amino acid length distribution of the TRB CDR3 rearrangements

**Figure 7:**
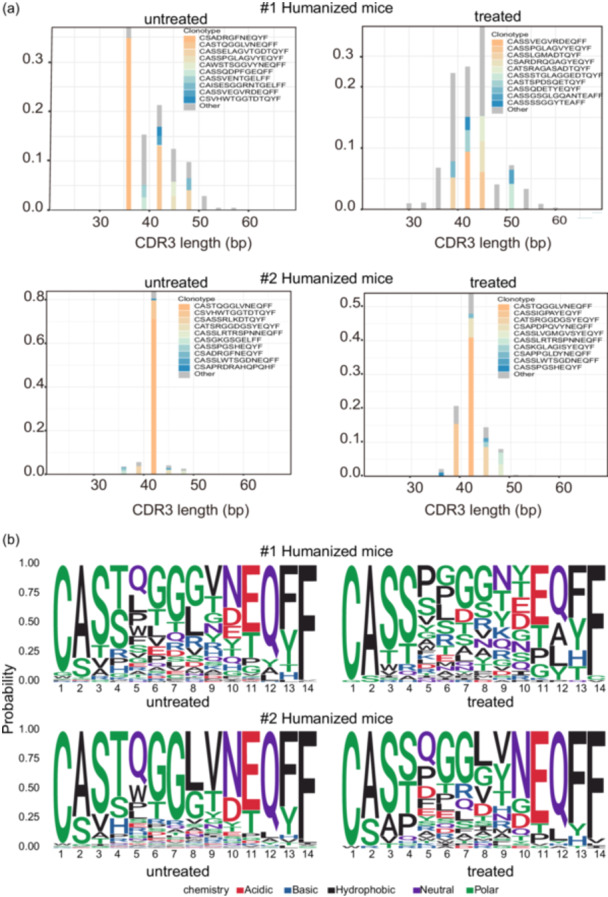
CDR3 length distribution and diagram of CDR3 motifs. (a) The distribution pattern of CDR3 length was analyzed for humanized mice. The amino acid (AA) length of TCR β‐chain V–J rearrangements in the CDR3 repertoire was between 35 and 50, and the highest peak was 45 AA for humanized mouse #1 and 42 AA for humanized mouse #2. (b) Motif analyses of the CDR3 regions showed different AA usage of T cells in female‐ and male‐derived PBMNC‐constructed humanized mice. Inactive cancer cell treatment led to a significant change in AA usage in the CDR3 repertoire. Bit height corresponds to the AA identity likelihood. Red—acidic AAs, blue—basic AAs, black—hydrophobic AAs, purple—neutral AAs, green—polar AAs. The more AAs presented in the same position, the larger the letter. Multiple letters indicate that there are different AAs in this position.

The range of CDR3 nucleotide lengths presented a normal distribution (Gaussian distribution) (Figure 7a), ranging between 35 bp and 45 bp in all groups. The untreated group had the highest frequency of CDR3 at a length of 36 bp (humanized mouse #1) and 42 bp (humanized mouse #2), whereas the treated group had the highest frequency of CDR3 at a length of 45 bp (humanized mouse #1) and 42 bp (humanized mouse #2) (Figure 7b). A Gaussian CDR3 AA length distribution pattern was also observed, and the highest peak of 14 AAs was distributed in the CDR3 repertoires (Figure 7b).

### 3.12 Quantification of tumor burden and survival for the humanized mice

**Figure 8:**
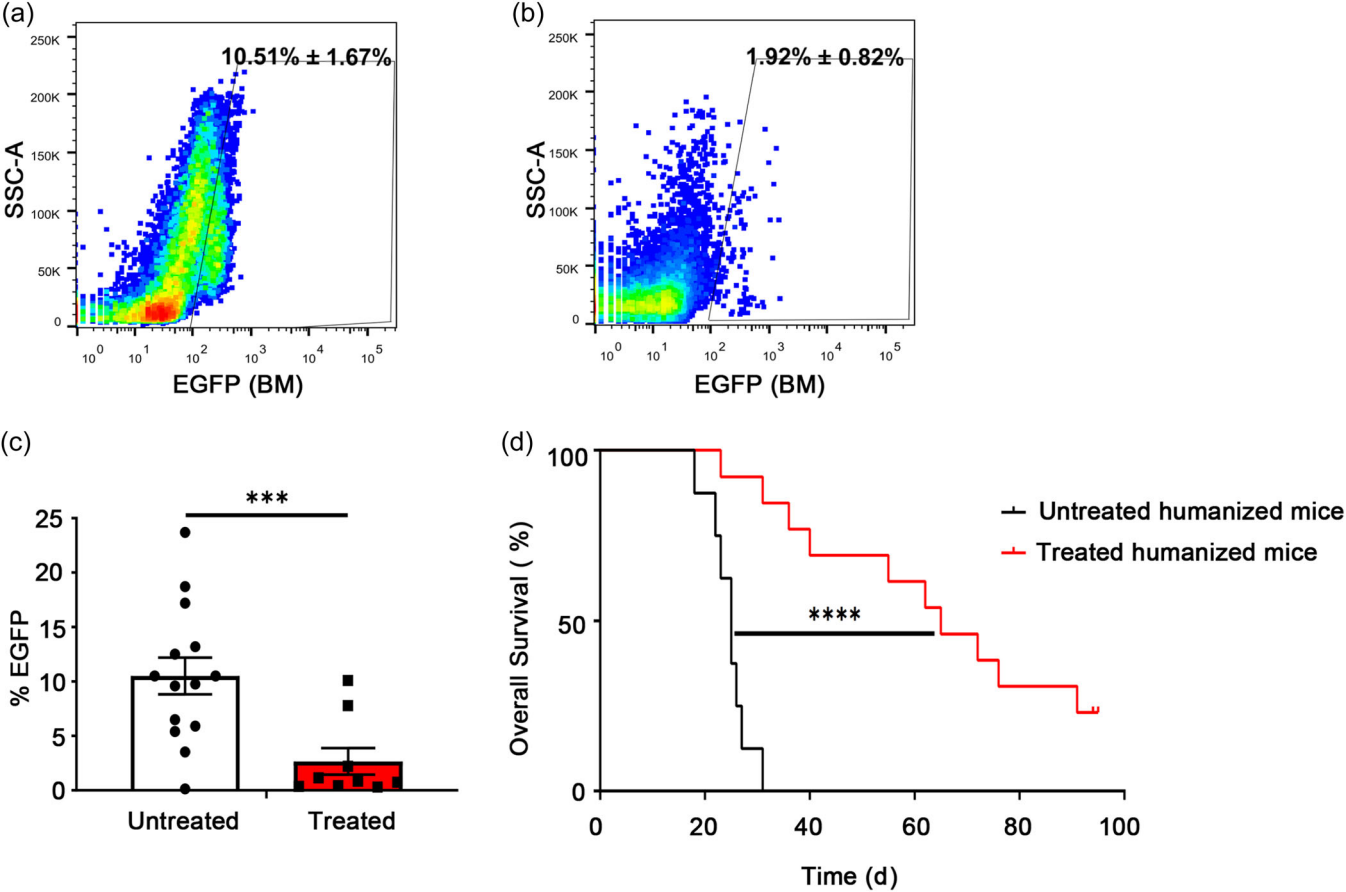
Inactive cancer cell inoculation treatment enabled mice to inhibit the growth of subsequently inoculated tumor cells. (a) Xenogeneic tumor cells with EGFP proliferated rapidly in NCG mice. (b) The growth of tumor cells was significantly inhibited in the humanized mice. (c) Cancer burden analysis was calculated. (d) Inactive cancer cell inoculation treatment efficiently prolonged the life span of the humanized mice. *N* = 20. ***p < 0.001; ****p < 0.0001.

To evaluate whether the inactive cancer cell infusion exerted an effect on the growth of tumor xenografts and the survival of mice, we further injected living 1 × 10^6^ NAML‐6‐EGFP and 1 × 10^6^ MDA‐MB‐231‐EGFP into the humanized mice after two consecutive inactive cancer cell infusions. Cancer burden was monitored by EGFP expression in organs 20 days after live cell injection. Representative flow cytometry plots are presented for EGFP expression in the bone marrow (Figure 8a,b), and statistical analysis of the data was conducted for the bone marrow, liver, and spleen (Figure 8c). The results showed that in untreated humanized mice, tumor cells were present in the bone marrow, liver, and spleen at a high proportion of 10.51% ± 1.67% (mean ± SEM) but were undetectable in peripheral blood (data not shown). This indicated that the bone marrow, liver, and spleen may represent niche environments in which the tumor cells can survive. Significantly, the number of tumor cells decreased in the immune inoculation groups to only 1.92% ± 0.82% (mean ± SEM), suggesting that in the humanized mouse model, immunization with inactive cancer cells may be effective at inhibiting the growth of tumors.

The survival curves of the mice were also evaluated (Figure 8d). The results demonstrated that the control NCG mice without any treatment died ~20 days after the transfusion of live tumor cells. However, survival of the humanized mice following immune inoculation was significantly prolonged, showing a higher survival rate than the control groups.

## 4 DISCUSSION

Several engineered T cells, such as CAR‐T cells, have been successfully applied in clinical practice and have demonstrated strong antitumor activities in hematological malignancy treatment but weak effects on solid tumors such as triple‐negative breast cancers [20, 21, 22].

Here, we applied steady‐state PBMNC‐derived 3D‐expanded cells to construct a humanized mouse model with human DCs, monocytes, and T cells, which are important tumor‐antigen‐presenting cells or tumor‐killing cells. Based on our model, we treated mice with inactive cancer cells on two occasions to stimulate an immune reaction. Human T cells in the untreated and treated humanized mice were collected, and TRB sequencing analysis was performed. By mapping to the reference sequences of the international ImMunoGeneTics information database (http://www.imgt.org), we obtained cancer‐specific responsive TRB CDR3 repertoires.

Higher TRB CDR3 diversity was detected in the treated humanized mice compared with the untreated groups, with an increasing fraction of unique clones in the peripheral blood after treatment. We inferred that the increase in CDR3 diversity might be caused by cancer cell‐derived neoantigen induction. Additionally, we observed the high expansion of CDR3 clones in the treated mice, and most expanded clones did not appear in the initial endogenous T‐cell pools but were newly produced after treatment. Considering the induced emergence of the TCR‐T cells, we speculated that these T cells harboring neoantigen‐induced novel TRB CDR3 sequences possessed cancer cell lytic activity. However, a more detailed investigation using a larger sample size for the cancer‐targeted T‐cell repertoire from humanized mice is warranted in future studies.

TCRs function via the recognition of foreign peptide presented by antigen‐presenting cells and subsequently induce an immune response [23, 24]. One of the characteristics of TCRs is diversity, as evidenced by the large repertoire of unique TCRs. The random and imprecise rearrangements of the V, D, and J segments of the CDR3 genes are an important mechanism to generate TCR diversity. In older patients or patients with immune disorders, the diversity of CDR3 dramatically decreases or the range of clonal sizes increases [25, 26]. The region of highest diversity in CDR3 is the key determinant of specificity in antigen recognition [24]. Therefore, the CDR3 profile provides specific insight into the antigen response after foreign antigen stimulation and is a critical indicator of the host immune status.

Some studies have reported that a decrease in T‐cell diversity often leads to an increase in susceptibility to various infections in older patients [25, 26, 27]. It has also been reported that immune diversity is higher in females than in males, which corresponds with life expectancy [28, 29]. In our study, we did detect higher diversity in the CDR3 profiles of females compared with males, although this finding should be confirmed with a larger sample size.

The strategy of screening cancer‐targeted CDR3 in humanized mice reveals alterations in host immunity in response to antagonism by cancer cells. The altered TRB CDR3 sequences can be identified and utilized for the design of engineered T cells, such as CAR‐T products. Strong heterogeneity is an important feature of cancer, especially following radiotherapy and chemotherapy [30, 31]. To date, it has been difficult to integrate these mutated, highly heterogeneous, cancer cell‐targeted segments into the design of engineered T cells. However, using our humanized mouse model constructed with person‐specific PBMNC‐derived 3D‐cultured cells, cancer‐targeted sequences of CDR3 are produced after inactive cancer cell stress in vivo that mimic natural antigen‐specific T‐cell production. Therefore, the highest diversity sequences of CDR3 are produced via the natural mechanism, with the affinity, avidity, antigen epitope location, and accessibility, as well as their effect on T‐cell functionality, all being naturally selected in vivo.

In this study, we injected active cancer cells into humanized mice to test the efficacy of TCR‐T cells rather than directly using defined cancer‐targeted T‐cell repertoires to treat tumors in mice. In fact, we tried to expand the cancer‐targeted T cells isolated from the humanized mice in vitro with human cytokines and factors (data not shown) but were unsuccessful. In the future, other approaches, such as synthetic biology or alternative culture systems will be tried to obtain specific and effective cancer‐targeted T cells in vitro.

Additionally, we did not analyze changes in T cells in organs other than the bone marrow, liver, and kidney, in the humanized mice, which are the known major niches for cancer cells. We assume that the dynamic changes in human T‐cell repertoires in the host will be consistent across all organs; however, this hypothesis and the mechanism involved should be studied in the future.

High‐throughput sequencing is an important platform for the investigation of cancer‐targeted rearrangement sequences in the CDR3 of TCR β‐chain repertoires [7, 32]. According to the CDR3 of β‐chain sequence profiling, we identified not only the variable sequence directing cancer antigens and clonal features at the molecular level but also cancer‐specific TCR‐T cells capable of targeting cancer cells. Accordingly, we revealed the diverse sequence changes and immunological features of CDR3 in human TCR‐T cells in the humanized mice by comparing CDR3 sequences before and after treatment with inactive cancer cells.

Our platform for constructing and screening cancer‐targeted TCR‐T cell repertoires has promising applications in the design of patient‐specific adoptive immunotherapy for some important malignancies.

## AUTHOR CONTRIBUTIONS

**Yulin Xu**: Data curation (equal); formal analysis (equal); investigation (equal); methodology (equal); project administration (equal); writing—review and editing (equal). **Wei Shan**: Validation (equal); visualization (equal); writing—original draft (equal). **Qian Luo**: Investigation (equal); methodology (equal). **Meng Zhang**: Resources (equal); supervision (equal); validation (equal); visualization (equal). **Dawei Huo**: Supervision (equal); validation (equal); visualization (equal). **Yijin Chen**: Data curation (equal); investigation (equal); writing—original draft (equal). **Honghu Li**: Investigation (equal); writing—original draft (equal). **Yishan Ye**: Supervision (equal); validation (equal); visualization (equal). **Xiaohong Yu**: Supervision (equal); validation (equal). **Yi Luo**: Supervision (equal); validation (equal); visualization (equal). **He Huang**: Supervision (equal); validation (equal); visualization (equal).

## CONFLICT OF INTEREST STATEMENT

The authors declare no conflict of interest.

## ETHICS STATEMENT

The animal study was reviewed and approved by the Animal Experimental Ethical Inspection of the First Affiliated Hospital, College of Medicine, Zhejiang University (Reference Number: 2020‐1167).

## INFORMED CONSENT

Ethical approval for the use of PB was obtained from the Ethics Committees of the First Affiliated Hospital of Zhejiang University School of Medicine (Expedition review No. 0465 in 2023).

## Supporting information

Supporting information.

Supporting information.

Supporting information.

Supporting information.

Supporting information.

Supporting information.

## Data Availability

The data from this study are available on request from the corresponding author.
