# Supplementary figures and images for: Establishment of a humanized mouse model using steady‐state peripheral blood‐derived hematopoietic stem and progenitor cells facilitates screening of cancer‐targeted T‐cell repertoires
Source: Cancer Innov. 2024 Apr 15;3(3):e118. doi: 10.1002/cai2.118 (PMC11212321; doi:10.1002/cai2.118)

## Total frequency of the top 10 TCRB clones

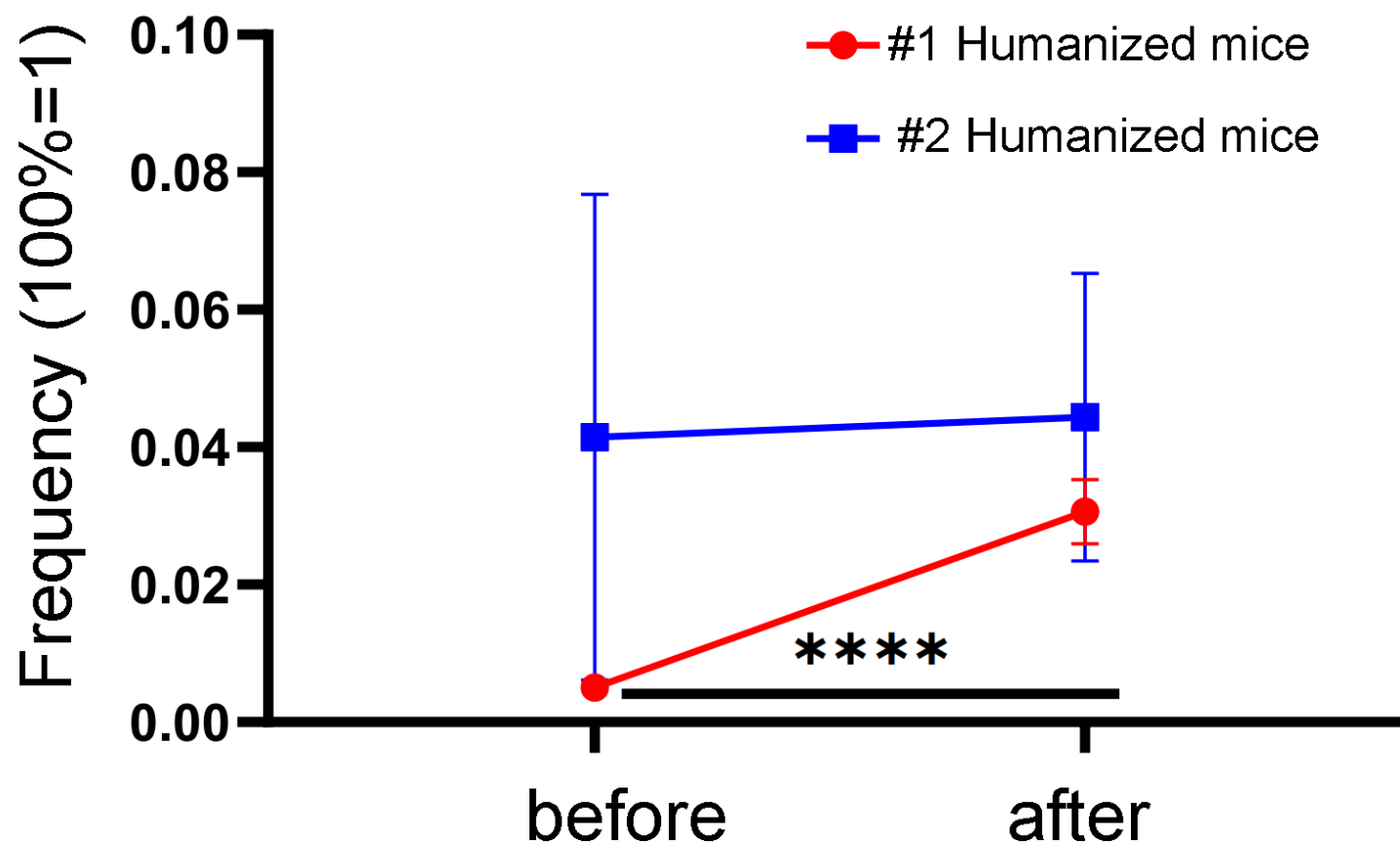

Supplement: Supplementary file 1 — Supporting information. [file CAI2-3-e118-s005.pdf]
